# S14G-Humanin ameliorates ovalbumin-induced airway inflammation in asthma mediated by inhibition of toll-like receptor 4 (TLR4) expression and the nuclear factor κ-B (NF-κB)/early growth response protein-1 (Egr-1) pathway

**DOI:** 10.18632/aging.204874

**Published:** 2023-07-14

**Authors:** Bo Su, Ran Li, Fuxing Song, Min Liu, Xianjun Sun

**Affiliations:** 1 Department of Pediatrics, Jinan City People’s Hospital, Jinan 250102, Shandong, China

**Keywords:** OVA-induced asthma, S14G-Humanin, inflammation, NF-κB, Egr-1

## Abstract

Asthma is a chronic inflammatory disease with a high morbidity rate in children and significantly impacts their healthy growth. It is reported that Th2 cell-mediated airway inflammation and activated oxidative stress are involved in the pathogenesis of asthma. S14G-humanin (HNG) is a derivative of Humanin with higher activity. The present study proposes to explore the potential treating property of HNG on asthma. An asthma model was constructed in mice using ovalbumin (OVA), the mice were treated with 2.5 mg/kg and 5 mg/kg HNG for 16 days. Dramatically increased lung weight index, elevated number of monocytes, eosinophils, and neutrophils, promoted production of Th2 cytokines including interleukin-4 (IL-4), interleukin-5 (IL-5), and interleukin-13 (IL-13), and severe histological pathology were observed in OVA-challenged mice, all of which were extremely alleviated by 2.5 mg/kg and 5 mg/kg HNG. Furthermore, the increased malondialdehyde (MDA) level and declined superoxide dismutase (SOD) activity in OVA-challenged mice were abolished by 2.5 mg/kg and 5 mg/kg HNG. Lastly, the upregulated TLR4, p-NF-κB p65, and early growth response 1 (Egr-1) in lung tissues of OVA-challenged mice were pronouncedly downregulated by 2.5 mg/kg and 5 mg/kg HNG. Collectively, our data suggested that HNG ameliorated airway inflammation in asthma partially due to NF-κB and Egr-1-mediated responses.

## INTRODUCTION

Asthma is a common chronic inflammatory disease with high difficulty to be completely cured and an increasing incidence rate. The global morbidity of asthma is about 1-30%, and relevant studies show that global asthma patients have exceeded 300 million [1], amongst which the proportion of children increases annually, with serious effects on their healthy growth [2]. The main clinical characteristics of asthma include coughing, wheezing, chest tightness, shortness of breath, and variable airflow restriction, accompanied by pathological changes such as the chronic inflammatory response of the airway and abnormal apoptosis, shedding, and repair disorders of epithelial cells [3]. It is of great significance to better summarize the pathogenesis of asthma and explore its treatment strategies. At present, the specific pathogenesis of asthma remains unclear. However, airway inflammation is regarded as the most fundamental pathological change. Components involved in airway inflammation in asthma include eosinophils, mast cells, basophils, neutrophils, helper T cells (Th cells), dendritic cells, and a variety of cytokines [4, 5], amongst which T cells play an important role. Th1 and Th2 cells are the main subtypes of Th cells. Under a normal physiological state, Th1/Th2 is in a dynamic balance. However, Th1/Th2 immune imbalance, Th1 immune suppression, and Th2 immune hyper-reaction are reportedly important in the pathogenesis of asthma [6]. Studies have confirmed that the production of Th2 cytokines can be promoted by Toll-like receptors (TLRs) through the signaling transduction pathway of airway epithelial cells, leading to airway inflammation [7]. Also, toll-like receptors (TLRs) are the ‘gate keepers’ of the immune system in humans and other animals to protect the host from invading bacteria, viruses, and other microorganisms [8]. TLR4 is reported to initiate a downstream inflammatory cascade by activating the NF-κB pathway [9] and mediating the lipopolysaccharide (LPS)- induced inflammatory response in asthmatic models [10]. In addition to inflammation, studies have shown that compared to non-asthmatic patients, systemic oxidative stress is dramatically activated in asthmatic patients, and exacerbation of acute asthma further increases the degree of oxidative load [11]. Under various stimuli such as allergens, airway oxidative stress is activated and antioxidant capacity is weakened, resulting in airway structural damage and metabolic changes, which promote the development of asthma [12]. Early growth response-1 (Egr-1), a zinc finger transcription factor, could be induced by a variety of receptors, growth factors, and signaling pathways. Recently, Egr-1 has been reported to act to suppress the epidermal growth factor receptor-mediated pathway and vascular muscularization, fibrosis, and airway hyperresponsiveness in the absence of inflammation [13]. Additionally, Egr-1 gene polymorphisms have been associated with total IgE and atopy in asthmatic children [14]. Interestingly, the interaction between Egr-1 and the NF-κB pathway has been found in the initiation and development of lung inflammation [15]. Therefore, TLR4/NF-κB- and Egr-1- mediated inflammatory response and oxidative stress might be important therapeutic targets for asthma.

Humanin is an endogenous small molecule polypeptide derived from mitochondria. It consists of 24 amino acids and was first discovered in the brain tissue of Alzheimer's disease patients in 2001 [16]. Humanin is widely expressed in the brain, blood vessel walls, testicles, and intestines, and has been reported to play a significant biological role in age-related diseases, such as longevity, metabolism, and inflammation [17]. S14G-humanin (HNG) is a derivative of Humanin, the activity of which is 1000 times higher than Humanin. It is recently reported that HNG shows a promising inhibitory effect on TLR4/NF-κB- mediated inflammation [18] and oxidative stress [19], and it has also been reported to inhibit NLRP3 activation induced by UV-B in retinal endothelial cells through mitigating the activity of Egr-1 [20]. HNG also plays an important role in inhibiting the expression of pro-inflammatory cytokines, including TNF-α, IL-1β, IL-6, and MCP-1 in a murine stroke mode and reducing the attachment of monocytes to bEnd.3 cells through modulating the NF-κB signaling pathway. However, it is unknown whether HNG possesses a protective effect on asthma. The present study aims to investigate the effects of HNG on airway inflammation in ovalbumin-induced asthma mice models and explore the underlying mechanism.

## MATERIALS AND METHODS

### Animals, modeling, and grouping

C57 mice were obtained from Shanghai Slac Laboratory Animal Company Limited (Co. Ltd) and the asthma model was established in mice using ovalbumin (OVA). In brief, mice (n=8 for each group) were sensitized with 10 mg OVA (#S7591, Sigma-Aldrich, USA) and 1 mg aluminum hydroxide was dissolved in 500 μL saline intraperitoneally on day 0 and day 7. From day 14 to 16, mice were exposed to OVA nebulization in saline solution (1% w/v) for 30 min. Each animal was challenged using an ultrasonic nebulizer with a flow rate of 1 ml/min. Animals were divided into 4 groups (10 mice/group): Vehicle, OVA, OVA+ 2.5 mg/kg HNG (#H6161, Sigma-Aldrich, USA), and OVA+ 5 mg/kg HNG. Animals in the OVA group were treated according to the procedure described above. In the vehicle group, healthy mice were sensitized with 1 mg aluminum hydroxide on day 0 and day 7, followed by being treated with saline aerosol for 30 min from day 14 to 16. Mice in the OVA+ 2.5 mg/kg HNG and OVA+ 5 mg/kg HNG group were treated (i.p) daily with 2.5 mg/kg and 5 mg/kg HNG from day 0 to day 16 [21], respectively.

### Lung weight index determination

Briefly, after a 16-day treatment, animals were weighed, executed and lung tissues were extracted for weighing. Then, the lung weight/body weight ratio was calculated to determine the lung weight index.

### Lung oxidative stress biomarkers determination

The content of MDA and the activity of SOD in lung tissues were examined to evaluate the profiles of oxidative stress. A commercial kit (CAT#: E-BC-K028-M, Elabscience, USA) was utilized to detect the MDA content in lung tissues using the TBA method according to the instructions of the kit. The activity of SOD in lung tissues was determined with an EnzyChromSuperoxide Dismutase Assay Kit (CAT#: ESOD-100, BioAssay Systems, USA) according to the instructions of the kit.

### Collection of bronchoalveolar lavage fluid (BALF) and quantification of differential WBCs counts

After the animals were sacrificed, endotracheal intubation was performed and the left lung was ligated at the left principal bronchus, followed by lavage of the right lung with 1.5 mL physiological salt water 3 times. The cell supernatant of each group was collected after centrifugation at 1500 rpm for 5 minutes. The sediment was collected for cell counting with a blood cell counting plate.

The hemocytometer was used to count the total monocytes in the BALF. Flow cytometry was conducted to determine the number of eosinophils and neutrophils in the BALF. Ly-6G is a marker of neutrophil-derived EVs [22], and CCR3 is a surface marker of eosinophils [23]. Total cells were blocked with an Fc blocker (Cat#: 553141, BD Biosciences, USA) for 10 min. The antibody against Ly6G (Cat# 553128, BD Biosciences, USA), CCR3 (Cat# 559923, BD, Biosciences, USA) and CD3/CD4 (Cat#555276/550280, BD Biosciences, USA) was added to be incubated separately to select eosinophils, neutrophils, and lymphocytes for half an hour at 4° C, followed by loading to the flow cytometer (BD Biosciences, USA) for the identification of cell number of neutrophils and eosinophils.

### Lung histopathological examination

Lung tissues were washed with water 2 times, followed by dehydration with 70%, 80%, and 90% ethanol solution, successively. Then, the xylene was used for dehydration, followed by embedding for 60 min and then sliced. Sections were roasted, dewaxed, hydrated, immersed in distilled water, and dyed in hematoxylin aqueous solution for 3min, followed by being differentiated with hydrochloric acid ethanol differentiation solution for 15 s. After being rinsed, slides were dyed with eosin (Cat#, 15086-94-9, Sigma-Aldrich, USA) for 3 min, followed by taking images with the inverted microscope (Zeiss, Germany). 3 fields in each lung specimen were examined. The severity of peribronchial inflammation was graded semi-quantitatively for the following features [24]: 0, normal; 1, few cells; 2, a ring of inflammatory cells 1 cell layer deep; 3, a ring of inflammatory cells 2–4 cells deep; and 4, a ring of inflammatory cells of 4 cells deep.

### Enzyme-linked immunosorbent assay

The release of Th2 cytokines IL-4 (#D711052, Sangon Biotech, China), IL-5 (#D711087, Sangon Biotech, China), and IL-13 (#D711053, Sangon Biotech, China) in the BALF was assessed using ELISA. After balancing for 1 h, the required lath was picked out and the standards or samples were added. The samples were incubated with 50 μL biotin-labeled antibody at 37° C for 30 min and then washed 3 times. Next, 100 μL horseradish peroxidase (HRP) labeled detection antibody was introduced to each well, followed by sealing and incubation at 37° C for 30min. After discarding the liquid and 3 washes, 50 μL substrate A and B were added and incubated at 37° C for 15min in the dark. After adding 50μL stop solution to each well, the optical density (OD) value of each well was measured at 450nm wavelength within 15min using a microplate analyzer (Molecular Devices, USA).

### Western blot analysis

The BCA kit (Solarbio, China) was used for the quantification of protein in lung tissues, followed by being separated with a 12% SDS-PAGE and further transferred from the gel to the PVDF membrane. After blocking using 5% skim milk, the membrane was added with the primary antibody against TLR4 (Cat# MBS540468, 1:1000, Affinity, Australia), p-NF-κB (Cat# ab16502, 1:1000, Abcam, UK), Egr-1 (Cat#4153, 1:1000, Cell Signaling Technology, USA), and β-actin (Cat#AF7018, 1:5000, Affinity, Australia). The second antibody (1:3000, Cell Signaling Technology, USA) was then introduced to be incubated for 90 min. Finally, the ECL reagent was added to expose the bands. We scanned the western blot results and selected target bands. Then the sum optical density of the bands was quantified using the Kodak Digital Science 1D software (Eastman Kodak Company, USA) and exported for statistical analysis.

### Statistical analysis

Obtained data were presented as mean±SD and were analyzed with the GraphPad software. The ANOVA method with Tukey’s post hoc test was applied for comparison. *P<0.05* was regarded as a significant difference.

### Consent to publication

All the authors have read and approved the final submission of this study.

### Data availability statement/availability of data materials

The data that support the findings of this study are available from the corresponding author upon reasonable request.

## RESULTS

### HNG reduced lung weight index in the OVA-induced murine model of chronic asthma

**Figure 1:**
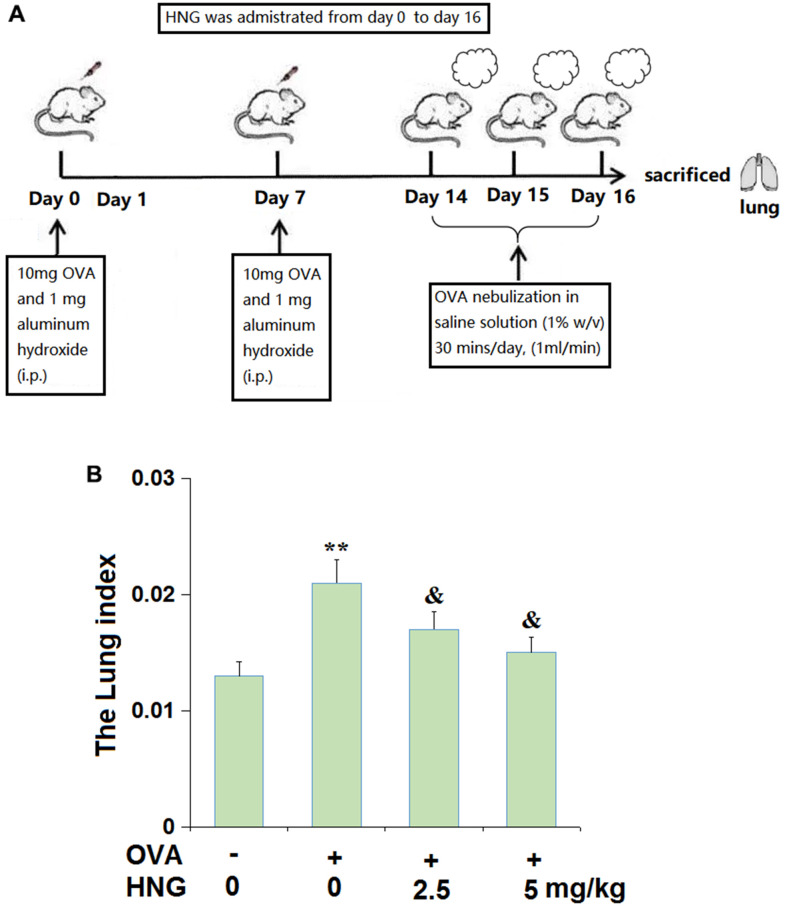
**S14G-Humanin (HNG) reduced lung weight index in ovalbumin (OVA)- induced murine model of chronic asthma.** (**A**) The outline of the experimental protocol; (**B**) The Lung index was assayed (**, *P<0.01* vs. vehicle group; &, *P<0.05* vs. asthma models).

The outline of the experimental protocol is displayed in Figure 1A. OVA-induced inflammation features significant lung edema due to severe lung injury, so, the lung weight index was determined in this study. After treatment, animals were sacrificed to calculate the lung weight index. We found that the lung weight index (Figure 1B) in OVA-challenged mice was extremely elevated from 0.013 to 0.021, then dramatically declined to 0.017 and 0.015 by 2.5 mg/kg and 5 mg/kg HNG, respectively.

### HNG ameliorated oxidative stress in the OVA- challenged murine model of chronic asthma

**Figure 2:**
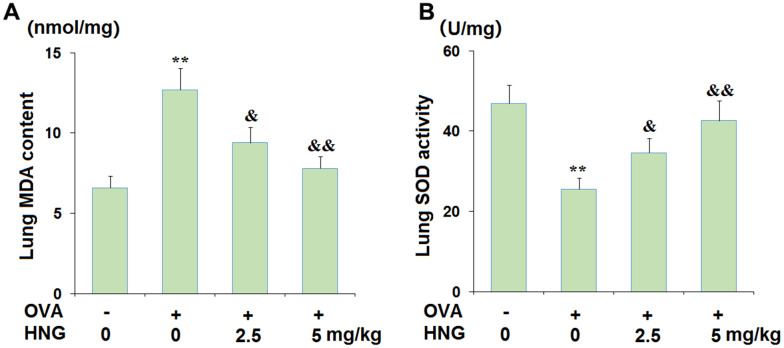
**S14G-Humanin (HNG) ameliorated oxidative stress in ovalbumin (OVA)- challenged murine model of chronic asthma.** (**A**) Lung MDA content; (**B**) Lung SOD activity (**, *P<0.01* vs. vehicle group; &, &&, *P<0.05*, 0.01 vs. asthma models).

Oxidative stress is reportedly observed during the pathogenesis of asthma [25], which was investigated in each animal. Compared to the vehicle group, the pulmonary MDA content (Figure 2A) was greatly increased from 6.6 nmol/mg protein to 12.7 nmol/mg protein in OVA-challenged mice but dramatically reduced to 9.4 and 7.8 nmol/mg protein by 2.5 mg/kg and 5 mg/kg HNG, respectively. Furthermore, the pulmonary SOD activity (Figure 2B) in the vehicle, OVA, OVA+ 2.5 mg/kg HNG, and OVA+ 5 mg/kg HNG groups was 46.9, 25.6, 34.6, and 42.6 U/mg wet tissue, respectively. These data suggest that the OVA-induced oxidative stress in mice was ameliorated by HNG.

### The effects of HNG on differential cell counts in BALF

**Figure 3:**
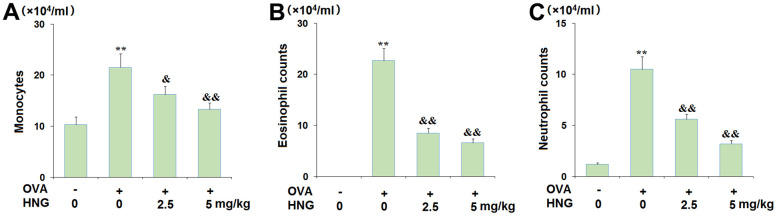
**The effects of S14G-Humanin (HNG) on differential cell counts in bronchoalveolar lavage (BAL) fluid.** Mice were sacrificed 24 h after the final OVA challenge, and BAL cells were isolated. (**A**) Monocytes; (**B**) Eosinophil counts; (**C**) Neutrophil counts (**, *P<0.01* vs. vehicle group; &, &&, *P<0.05*, 0.01 vs. asthma models).

Subsequently, the infiltration of inflammatory cells in the BALF of each animal was investigated. The total monocytes (Figure 3A) in the vehicle, OVA, OVA+ 2.5 mg/kg HNG, and OVA+ 5 mg/kg HNG groups were 10.3×10^4^/mL, 21.5×10^4^/mL, 16.2×10^4^/mL, and 13.3×10^4^/mL, respectively. The number of eosinophils (Figure 3B) in OVA-challenged mice was extremely increased from 0 to 22.7×10^4^/mL but greatly decreased to 8.5×10^4^/mL and 6.6×10^4^/mL by 2.5 mg/kg and 5 mg/kg HNG, respectively. Moreover, the neutrophil counts (Figure 3C) in the vehicle, OVA, OVA+ 2.5 mg/kg HNG, and OVA+ 5 mg/kg HNG groups were 1.2×10^4^/mL, 10.5×10^4^/mL, 5.6×10^4^/mL, and 3.2×10^4^/mL, respectively. These results reveal that the infiltration of inflammatory cells in the BALF of OVA-challenged mice was inhibited by HNG, but as shown in Figure 3D, there was no significant difference between the groups.

### HNG inhibited the expression of the Th2 cytokines IL-4, IL-5, and IL-13 from BALF

**Figure 4:**
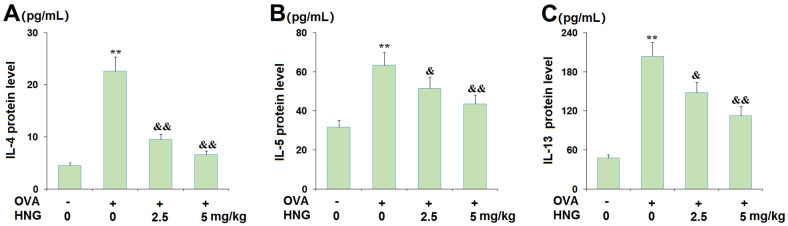
**S14G-Humanin (HNG) inhibited the expression of the Th2 cytokines interleukin-4 (IL-4), interleukin-5 (IL-5), and interleukin-13 (IL-13) from bronchoalveolar lavage (BAL) fluid.** (**A**) IL-4; (**B**) IL-5; (**C**). IL-13 (**, *P<0.01* vs. vehicle group; &, &&, *P<0.05*, 0.01 vs. asthma models).

Severe inflammation in BALF is regarded as an important pathological change in asthma. The level of Th2 cytokines was investigated. We found that the production of IL-4 (Figure 4A) was greatly elevated from 4.5 pg/mL to 22.6 pg/mL by the challenge with OVA, then dramatically reduced to 9.5 and 6.6 pg/mL by 2.5 mg/kg and 5 mg/kg HNG, respectively. In addition, the release of IL-5 (Figure 4B) in the vehicle, OVA, OVA+ 2.5 mg/kg HNG, and OVA+ 5 mg/kg HNG groups was 31.6, 63.3, 51.5, and 43.6 pg/mL, respectively. Moreover, compared to the vehicle, the level of IL-13 (Figure 4C) was promoted from 47.6 pg/mL to 203.6 pg/mL in OVA-treated mice but reduced to 147.9 and 112.4 pg/mL by the administration of 2.5 mg/kg and 5 mg/kg HNG, respectively. These data indicate that the release of Th2 cytokines in OVA-challenged mice was repressed by HNG. However, HNG alone has no effect on the expression of Th2 cytokines IL-4, IL-5, IL-13 (Supplementary Figure 1).

### HNG improved pathological changes in the OVA-induced murine model of chronic asthma

**Figure 5:**
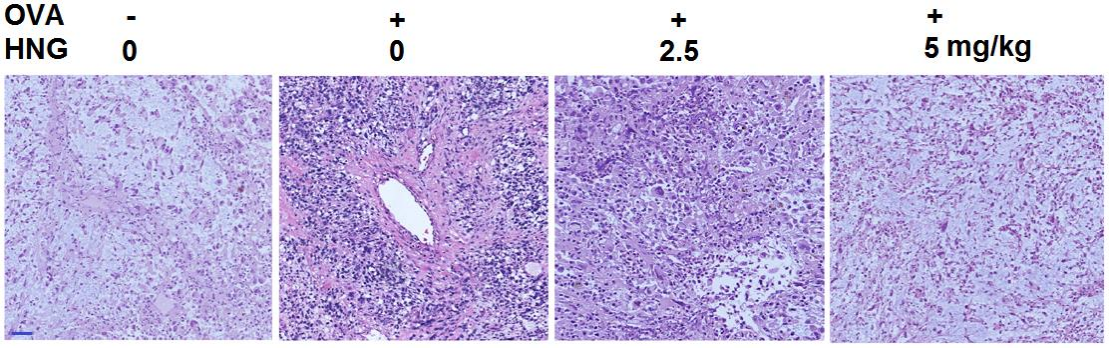
**S14G-Humanin (HNG) improved pathological changes in ovalbumin (OVA)- induced murine model of chronic asthma.** Hematoxylin and eosin (H&E) staining of lung tissue. Scale bar, 200 μm.

As illustrated in Figure 5, in the vehicle group, no degeneration, necrosis or shedding was observed in alveolar epithelial cells, with intact lung septum, alveolar cavity, bronchial cavity, and bronchial wall. In the OVA group, the pulmonary lobular structure was destroyed, alveolar epithelial cells were exfoliated, inflammatory cell infiltration and alveolar wall congestion were observed in 1/3 of the lung tissues, and the thickness of bronchial smooth muscle was significantly increased. After treatment with 2.5 mg/kg and 5 mg/kg HNG, the degree of tissue inflammation and infiltration of inflammatory cells in lung tissues was significantly alleviated, with milder pathological changes.

### HNG prevented the activation of TLR4/NF-κB and Egr-1 signaling

**Figure 6:**
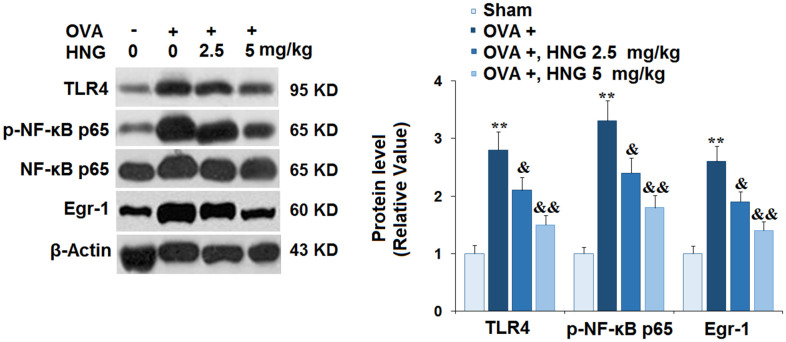
**S14G-Humanin (HNG) prevented the activation of toll-like receptor 4/ nuclear factor κ-B (TLR4/NF-κB) and early growth response-1 (Egr-1) signaling.** The levels of TLR4, p-NF-κB p65, and Egr-1 were measured using western blot analysis (**, *P<0.01* vs. vehicle group; &, &&, *P<0.05*, 0.01 vs. asthma models).

TLR4/NF-κB [26] and Egr-1 signaling [27] are reportedly involved in the pathogenesis of airway inflammation. We found that TLR4, p-NF-κB p65, and Egr-1 (Figure 6) were dramatically upregulated in OVA-challenged mice, then greatly repressed by 2.5 mg/kg and 5 mg/kg HNG. Consistently, HNG reduced the expression of TLR4 and Egr-1 at the mRNA levels (Supplementary Figure 2). These results suggest that the activation of TLR4/NF-κB and Egr-1 signaling in OVA-challenged mice was abolished by HNG.

## DISCUSSION

When the Th2 immune response is hyperactive, the secretion of Th2 cytokines, such as IL-4, IL-5, and IL-13 is extremely elevated, and they participate in the systemic immune response. IL-4 is secreted by activated Th2 cells and plays an important role in airway inflammation in asthma [28, 29], It induces the maturation of B lymphocytes to produce IgE under the synergistic effect of IL-5 [30]. IgE further binds with high-affinity receptors on the surface of B cells to promote the degranulation of B lymphocytes to release active substances such as leukotriene and histamine, leading to spasms of airway smooth muscle and increased airway reactivity [31–33]. These pathological changes contribute to the development of clinical symptoms of acute attack in asthma patients and even life-threaten bronchospasm in severe cases. IL-13 is mainly secreted by Th2 cells and partly produced by mast cells or monocytes. Receptors of IL-13 are widely located in a variety of cells, including airway epithelial cells [34, 35]. In the present study, severe pathological changes, including elevated lung weight index, infiltration of inflammatory cells, and histological pathology, were observed in OVA-challenged mice, similar to the description by Abdelaziz [36]. After treatment with HNG, the lung weight index, inflammatory cell infiltration, and histological pathology were dramatically ameliorated, implying a potential protective property of HNG against OVA-induced chronic asthma in mice. Furthermore, the release of Th2 cytokines in OVA-challenged mice was greatly repressed by HNG, suggesting a regulatory function of HNG on the Th1/Th2 balance.

Reactive oxygen species (ROS) are produced during regular metabolism. The production of ROS and antioxidants, such as SOD and glutathione peroxidase (GSH-Px), is relatively balanced to maintain normal physiological function [37] Under oxidative stress, increased ROS production and/or decreased antioxidant defense ability disrupt the balance, resulting in oxidative damage and aggravated inflammation [38]. Activated oxidative stress was observed in the OVA-induced murine model of chronic asthma in the present study, consistent with results reported by Faris Alrumaihi [39]. After administration of HNG, oxidative stress was alleviated, suggesting the function of HNG might be associated with ameliorated oxidative damage. Our future work on the regulatory mechanism of HNG on oxidative stress will be conducted to confirm the protective function of HNG against asthma.

NF-κB is one of the most studied transcription factors and was first identified as a regulator of the κB light chain gene expression in B lymphocytes about 30 years ago [40]. Nowadays, NF-κB signaling is widely recognized as a typical pro-inflammatory pathway for the production of pro-inflammatory cytokines, such as IL-1, TNFα, IL-4, IL-5, and IL-13 [41]. Currently, activation of NF-κB is known to regulate the expression of more than 500 genes [42] and the disorder of NF-κB signaling is closely related to the pathophysiology of multiple diseases, including bronchial asthma [43].

During the development of asthma, NF-κB is recruited by TLR4 to induce the production of several pro-inflammatory factors in the airway wall [44]. Early growth response factor-1 (Egr-1) is an important transcriptional factor involved in the development of cell proliferation and differentiation [45]. There is a growing body of evidence implying that Egr-1 was elevated in the pathogenesis of acute lung injury [46], and was considered a crucial gene in the activation process of the NF-κB pathway in lung inflammation [15]. Recent studies reveal that Egr-1 participates in the development of inflammatory reactions in human bronchial epithelial cells [47]. However, the effects of Egr-1 on asthma have been less reported. Zhang and colleagues have recently found that HNG inhibited LPS-induced inflammatory response in human dental pulp cells through the TLR4/MyD88/NF-κB pathway [18], which is consistent with our results. Our preliminary data indicate that the activation of TLR4/NF-κB and Egr-1 signaling in the OVA-induced murine model of chronic asthma was dramatically abolished by HNG, implying that the protective function of HNG against asthma might be closely associated with the inhibition of TLR4/NF-κB and Egr-1 signaling. However, which pathway or whether both pathways are responsible for the regulatory mechanism of HNG remains unclear. In future work, the functional mechanism of HNG will be verified in TLR4/NF-κB- or Egr-1- activated bronchial epithelial cells.

Altogether, the present study provides a more comprehensive molecular explanation for the action of HNG on asthma treatment. More preclinical experimental models will be carried out in the future to validate the therapeutic effects of HNG on asthma.

## Supplementary Material

Supplementary Figures
