# Supplementary material for: S14G-Humanin ameliorates ovalbumin-induced airway inflammation in asthma mediated by inhibition of toll-like receptor 4 (TLR4) expression and the nuclear factor κ-B (NF-κB)/early growth response protein-1 (Egr-1) pathway
Source: Aging (Albany NY). 2023 Jul 14;15(14):6822–33. doi: 10.18632/aging.204874 (PMC10415557; doi:10.18632/aging.204874)
Supplement: Supplementary Figures [file aging-15-204874-s001.pdf]

SUPPLEMENTARY FIGURES

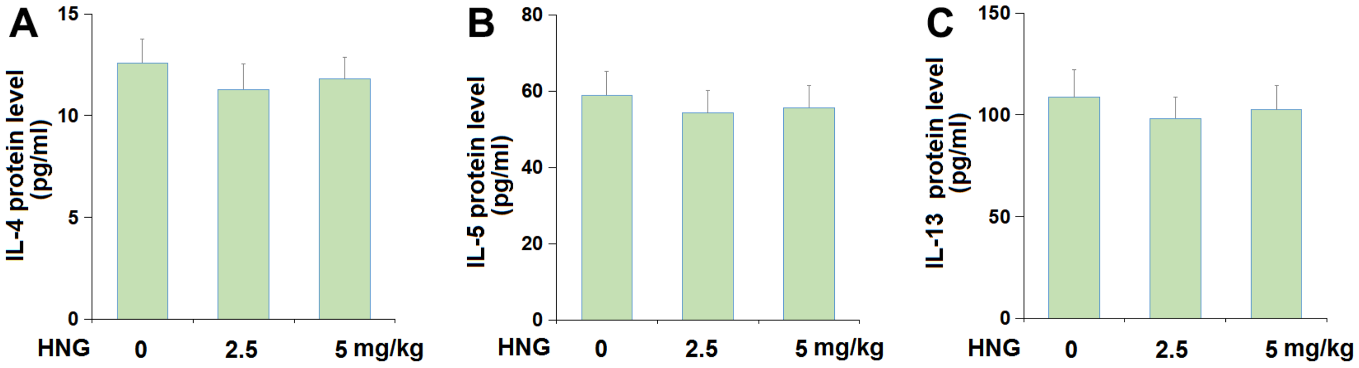

Supplementary Figure 1. S14G-Humanin (HNG) alone has no significant effect on the expression of the Th2 cytokines interleukin-4 (IL-4), interleukin-5 (IL-5), and interleukin-13 (IL-13) from bronchoalveolar lavage (BAL) fluid. (**A**) IL-4; (**B**) IL-5; (**C**) IL-13 in the BAL fluid.

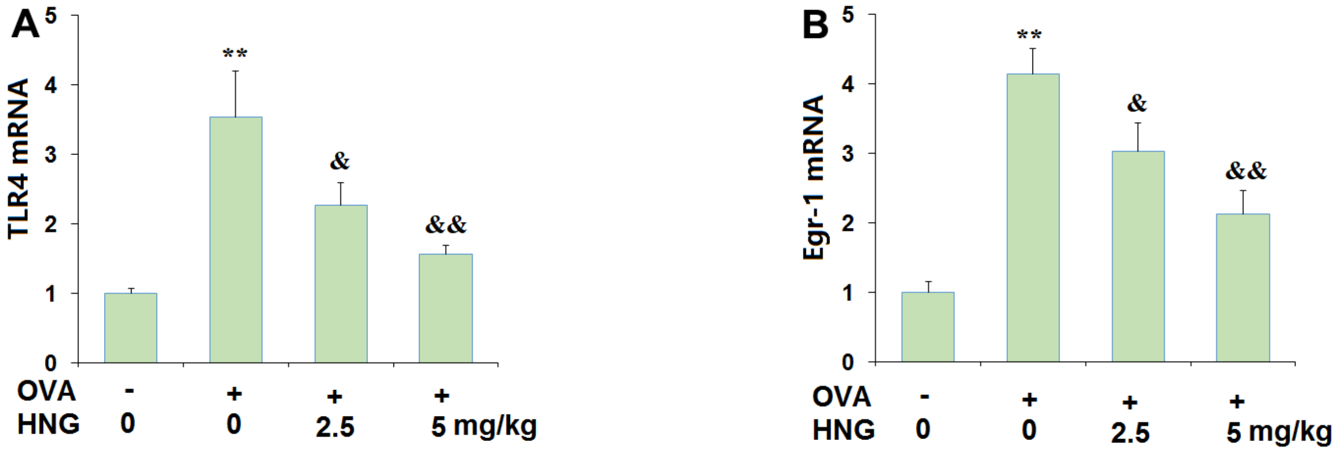

Supplementary Figure 2. S14G-Humanin (HNG) reduced the expression of toll-like receptor 4 (TLR4) and early growth response-1 (Egr-1). (**A**) mRNA of TLR4; (**B**) mRNA of Egr-1 (**, *P<0.01* vs. vehicle group; &, &&, *P<0.05*, 0.01 vs. asthma models).
